# Redox Balance Differentially Affects Biomechanics in Permeabilized Single Muscle Fibres—Active and Passive Force Assessments with the *Myorobot*

**DOI:** 10.3390/cells11233715

**Published:** 2022-11-22

**Authors:** Mena Michael, Larisa Kovbasyuk, Paul Ritter, Michael B. Reid, Oliver Friedrich, Michael Haug

**Affiliations:** 1 Institute of Medical Biotechnology, Department of Chemical and Biological Engineering, Friedrich-Alexander-University Erlangen-Nürnberg, Paul-Gordan-Str. 3, 91052 Erlangen, Germany; 2 Erlangen Graduate School in Advanced Optical Technologies (SAOT), Paul-Gordan-Str. 6, 91052 Erlangen, Germany; 3 College of Health & Human Performance, University of Florida, 1864 Stadium Road, Gainesville, FL 32611, USA

**Keywords:** redox balance, single muscle fibre, calcium sensitivity, passive stiffness, *MyoRobot*

## Abstract

An oxidizing redox state imposes unique effects on the contractile properties of muscle. Permeabilized fibres show reduced active force generation in the presence of H_2_O_2_. However, our knowledge about the muscle fibre’s elasticity or flexibility is limited due to shortcomings in assessing the passive stress–strain properties, mostly due to technically limited experimental setups. The *MyoRobot* is an automated biomechatronics platform that is well-capable of not only investigating calcium responsiveness of active contraction but also features precise stretch actuation to examine the passive stress–strain behaviour. Both were carried out in a consecutive recording sequence on the same fibre for 10 single fibres in total. We denote a significantly diminished maximum calcium-saturated force for fibres exposed to ≥500 µM H_2_O_2_, with no marked alteration of the pCa50 value. In contrast to active contraction (e.g., maximum isometric force activation), passive restoration stress (force per area) significantly increases for fibres exposed to an oxidizing environment, as they showed a non-linear stress–strain relationship. Our data support the idea that a highly oxidizing environment promotes non-linear fibre stiffening and confirms that our *MyoRobot* platform is a suitable tool for investigating redox-related changes in muscle biomechanics.

## 1. Introduction

It is well known that reactive oxygen species (ROS) play a crucial role in many cellular processes and influence the performance of muscles [1,2]. As free radicals, ROS shift the cell’s microenvironment towards a more oxidized state, which, in muscle fibres, was found to impact fatiguability, muscle damage, and active force generation [3,4,5,6]. Such deviation from its native state, or rather changes to the cell’s reductive or oxidative environment, were enclosed in the term *redox balance* [4]. Ever since then, studies have sought to unravel the effects, the origin, and the mechanisms of a shifted redox balance in muscle contraction. Even up to date, there is debate on how ROS are effectively produced, which species contributes to what extent, and what intracellular signalling pathways are involved in their action [1]. In that setting, hydrogen peroxide (H_2_O_2_) has attracted major interest, as it is naturally produced in mitochondria, an abundant organelle to be found in muscle cells [7,8]. Nowadays, the diverse effects attributed to H_2_O_2_ (e.g., altered Ca^2+^ sensitivity in intact fibres [9] or decreased maximum Ca^2+^-activated force in permeabilized fibres [5,10]) were found to be rather mediated by dismuted superoxide or hydroxyl radicals, both being produced in the presence of H_2_O_2_ [1,9]. Interestingly, more regular and frequent muscle activation accelerates the production of ROS [11], which leads to performance adaptation [4] but also cellular damage [12]. These effects also seem to increase with temperature [13].

So far, an oxidative redox balance due to H_2_O_2_ and/or related radicals was found to influence muscle fatigue, excitation–contraction coupling, maximum Ca^2+^ activated force, and the Ca^2+^ sensitivity in numerous studies [1,5,6,10]. While the findings from those studies improve our understanding of the redox balance in muscle, study outcomes vary (e.g., inhibiting effect of H_2_O_2_ to sarcoplasmic Ca^2+^-release [14] vs. a large insensitivity of the Ca^2+^-release towards externally applied H_2_O_2_ [6]). These differences, however, seem to relate to the use of either intact single muscle fibres or permeabilized muscle fibres.

From that perspective, particularly the influence of ROS on the Ca^2+^ sensitivity of the contractile apparatus is well studied. Intriguingly, ROS seem to have little effect on the Ca^2+^ sensitivity of the contractile apparatus in permeabilized muscle fibres [5,10,14], whereas intact fibre studies suggest a decreased Ca^2+^ sensitivity under oxidizing conditions (presence of H_2_O_2_) [1,4]. This is why it was hypothesized that ROS affect the Ca^2+^ sensitivity by pathways that seem to be absent/disrupted once the fibre is permeabilized and the cytosol is replaced by an externally applied solution [1]. The decrease in Ca^2+^ sensitivity is believed to occur from (cellularly) generated hydroxyl radicals or superoxide [9,15] that attack methionine or cysteine residues [16,17]. Therefore, ROS-related performance adaptations could at least be partially reversed by the anti-oxidant agent Dithiothreitol (DTT), predominantly in intact muscle fibres [1,4,18]. Additionally, in intact muscle fibres, DTT causes a further reduction in maximum force production [4], an effect that was not detected in permeabilized fibre preparations [3]. Consistently in permeabilized fibres, many studies have independently reported a decrease in the maximum Ca^2+^-activated force in the presence of DTT [5,10,15], which, in intact fibres, is suspected to originate from oxidized methionine residues [18]. Whether these potential interaction sites for DTT and H_2_O_2_ translate to the effects seen in permeabilized muscle fibres and how they align with the proposed pathways in intact muscle fibres remains yet to be resolved. Further, it is unclear from the literature how and to what degree these ROS-mediated changes impact the axial elasticity of the fibre, as an important parameter for damage prevention, structural memory, and the ability to resist large strains [19].

Therefore, we systematically assessed muscle mechanics at different redox balances in permeabilized muscle fibres during active contraction and passive force recordings with the automated *MyoRobot* biomechatronics platform [20]. We investigated the maximum Ca^2+^ activated force, the Ca^2+^ sensitivity of the contractile apparatus, and axial elasticity from stress–strain curves. All recordings were successively performed on the same fibre (sarcomere length (SL) adjusted in the range of 2.3–2.4 µm) in a fully automated sequence, both in the presence and absence of H_2_O_2_ (100 µM, 500 µM, 1000 µM), as well as in the reduced state (after 60 s of 1000 µM DTT incubation). Our findings of decreased Ca^2+^ activated force under oxidative stress (H_2_O_2_ presence) and unaltered Ca^2+^ sensitivity support previously proposed hypotheses and observations in permeabilized fibres. Based on these, we hypothesized that ROS exposure would increase the passive axial stiffness of muscle fibres, for which we carried out technically challenging passive stress–strain relationship recordings with our automated biomechatronics platform. Here, we indeed denote a significant fibre stiffening at high external concentrations of H_2_O_2_ that seem to affect the linear elastic deformation regime, potentially through inducing molecular stiffening. The technology presented here delivers consistent and reliable results and is, thus, well-suited to study the complex effects of ROS on the contractile properties of muscle.

## 2. Materials and Methods

### 2.1. Animal Handling and Single Fibre Preparation

All animal experiments were conducted in accordance with the approved animal experimentation guidelines at the Friedrich-Alexander-Universität Erlangen-Nürnberg (TS06/2016), the German regulations for the care of laboratory animals, and the guidelines of the Federation of European Laboratory Animal Sciences Associations. All animals for this study were male CS57BL/6 mice of approx. 30 g body weight and aged between 15–21 weeks. The samples were obtained through a tissue-sharing collaboration with local institutes. Anesthesia was induced by inhalation of 5% isoflurane, followed by cervical dislocation and subsequent cutting off of the hind limb. From here on, *M. extensor digitorum longus* (EDL) isolation was performed with physiological Ringer’s solution, which, after EDL isolation, was replaced by high K+ solution (HKS, see below) for manually tethering the single muscle fibres under a stereomicroscope (Olympus SZ-X7) using fine forceps (Dumont #5) and scissors (FST). HKS permanently depolarizes the membrane and inactivates Na+ channels after an initial contraction. During that procedure, the muscle was constantly pinned onto a Polydimethylsiloxane (PDMS, Sylgard, Dow Corning)-coated Petri dish under slight pre-stretch. Isolated single muscle fibres were then tied to two micro silk knots (each end) and transferred to the *MyoRobot* for fibre mounting in an HKS droplet. Each fibre was tied with one end to the pin of a force transducer (FT) and the other end to a voice–coil actuator (VC) before lowering it in the idle well underneath. Please note that prior to any other solution exposure, the single fibre was chemically permeabilized for 20 s in 0.01% (*w*/*v*) saponin. This permeabilization ensures complete diffusional access to the intracellular myoplasm, in particular, to clamp the myoplasmic free Ca^2+^ to any desired levels and to initially wash out endogenously produced ROS from the preparation. Additionally, since the Ca^2+^ solutions were highly EGTA-buffered, SR Ca^2+^ release would not affect the steady-state pCa-force data [21,22].

*High-Potassium Solution (HKS):* 140 mM K-glutamate, 1 mM MgCl_2_, 10 mM HEPES buffer, 1 mM EGTA, 10 mM glucose, pH: 7.0, osmolality: 275 mosmol/kg.

### 2.2. System Electronics and Software

The *MyoRobot*, described in more detail in [20], is a fully automated biomechatronics platform to assess a plethora of active and passive biomechanics properties. For this, it is equipped with a piezo-optical FT (TR5S, Myotronic UG, Heidelberg, Germany) and a linear VC actuator (CAL12-010-51-B5A, SMAC) acting as a counter pin for fibre mounting. A rack housing 32 individual wells to be filled with distinct bioactive solutions is actuated via a bi-axial linear stage configuration to allow automated exposure to different environmental conditions (see Active and Passive Force Assessment Protocols). The FT data are sampled at 100 Hz during each recording and read and displayed in a LabVIEW-written (National Instruments) user interface. The latter is likewise used to operate the entire setup digitally, as well as load and execute the biomechanics recording protocols. The rack, to whose solutions the sample is exposed, features a glass bottom with a miniaturized ∼25× magnifying optics system. This allows for capturing the morphological sample parameters, such as the SL and fibre diameter (cross section assumed as circular) online.

### 2.3. Bioactive Solutions

The solutions for fibre manipulation (activation or relaxation) were used and composed as given in [23]. Their summarized physiological purpose is mentioned in the table below (Table 1). In general, High Activating solution (HA) is an EGTA-buffered Ca^2+^-rich solution that activates troponin-C. High Relaxing solution (HR) is a Ca^2+^-free, EGTA-buffered solution to relax the fibre. Low Relaxing solution (LR) relies on the mild Ca^2+^ buffering agent HDTA and is likewise Ca^2+^ free. All of the control (CTRL) experiments were carried out in the absence of H_2_O_2_, while any recordings at oxidizing conditions were achieved by supplementing the base solutions with 100, 500, or 1000 µM H_2_O_2_. Reducing conditions were achieved by incubation in dithiothreitol (DTT) for 60 s before returning to the base solutions, free of H_2_O_2_.

### 2.4. Active and Passive Force Assessment Protocols

**Figure 1:**
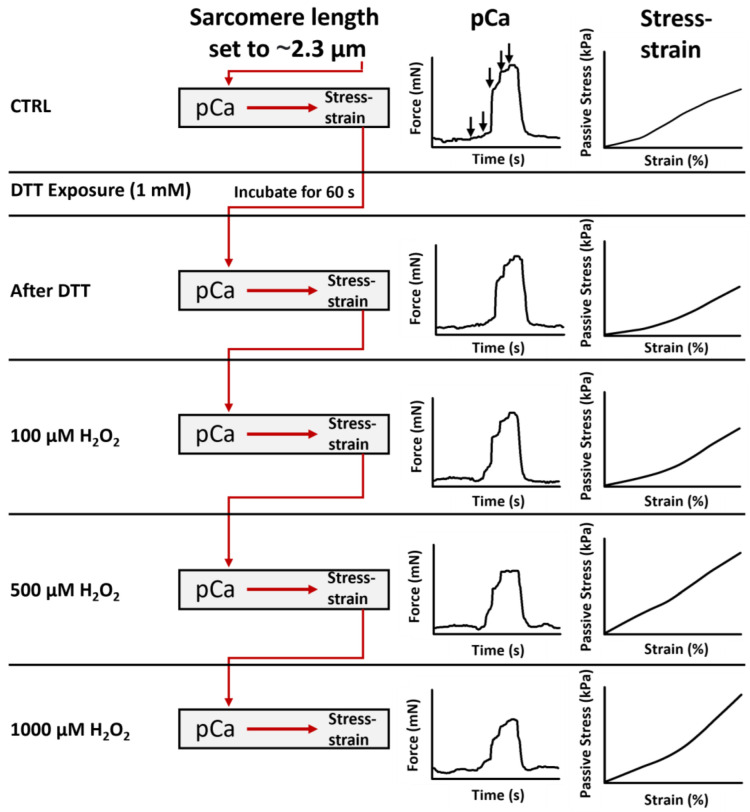
Sequence of recordings performed in one single muscle fibre. Each recording investigated five conditions: Control (CTRL), after 60 s of DTT exposure (1 mM), in 100 µM H_2_O_2_, in 500 µM H_2_O_2_ and in 1000 µM H_2_O_2_. For each condition, the single fibre was tested for active force generation in pCa-force curves and passive material properties in stress–strain recordings. While CTRL experiments represent the fibre’s native redox state, a 60 s DTT exposure (1 mM) shifts the redox balance towards a more reduced environment, whereas submersing the fibre in solutions with increasing H_2_O_2_ concentration produces a shift towards a more oxidizing environment. The vertical arrows indicate pCa solution exchanges. Axis are equally scaled for each respective recording protocol.

In the present study, we utilized our *MyoRobot* technology to study the effects of different redox environments on permeabilized single muscle fibre biomechanics. Being equipped with a 32-well rack allows our biomechatronics system to perform all active and passive force recordings under five different conditions (CTRL, DTT (reduced), 100 µM H_2_O_2_, 500 µM H_2_O_2_, and 1000 µM H_2_O_2_) in the same single fibre. Each experiment took approx. 3 h. The sequence of recordings is depicted in a flow chart in Figure 1.

First, single EDL muscle fibres were chemically permeabilized by immersion for 20 s in saponin 0.01% (*w*/*v*). The so-called ‘*skinned*’ fibre preparation is known to be very robust, and experimental sessions on a single fibre can robustly involve repetitive sets of complete pCa-force activation cycles [24,25]. Consequently, the fibre’s average sarcomere length (SL) was set to ∼2.3 µm by adjusting the position of the VC (equivalent to elongating the fibre), and its diameter was read out. Then, we recorded the pCa-force relationships and carried out stress–strain recordings under CTRL conditions after 60 s DTT incubation and in solutions supplemented with 100 µM H_2_O_2_, 500 µM H_2_O_2_, and 1000 µM H_2_O_2_, as given in Figure 1.

*Active Force—Ca^2+^ sensitivity of the contractile apparatus & maximum* Ca^2+^ activated force. Single fibres were exposed to wells with solutions of increasing Ca^2+^ concentration (correspondingly decreasing pCa values) to assess the Ca^2+^ sensitivity at the chemical–mechanical interface of troponin C. At a pCa value of 4.92 (Ca^2+^ saturated conditions), force generation was assumed to be maximum. Force generation followed a staircase-like pattern, as seen in Figure 1. The analysis included extracting the steady-state forces for each pCa step and plotting them as a pCa-force graph. Fitting a sigmoidal curve to the data ($y=\frac{10^{-bx}}{c^{b}+10^{-bx}}$, with x being pCa, and y being force, [26]) allowed for the extraction of the pCa_50_ value (−*log*_10_(*c*)) and the Hill parameter (b). Maximum Ca^2+^ saturated force was calculated according to the steady-state force at pCa 4.92.

*Passive Force—Stress–strain relationships* via *slow, linear fibre elongation.* The stress–strain relationships were recorded in LR solution to maintain relaxing conditions during the protocol. The VC elongated the fibre at an optimized velocity of 1 µm/s from 100% to 140% of its resting length (=40% strain). Knowing the force and the fibre diameter allowed us to convert forces to the more robust parameter *stress*, also taking the cross-sectional area into account. The maximum restoration stress was computed as the stress exerted at 40% strain. The axial stiffness was extracted from the force–elongation curves, as linear fits were applied to each section of 10% strain. The increase in the respective fit curve equals the passive axial stiffness [27].

## 3. Data Analysis

All of the data were analysed for normality by performing a Shapiro–Wilk test to determine the appropriate testing procedure. When a data set was not normally distributed, nonparametric tests were adopted. In this case, a Kruskal–Wallis test was performed based on the rank sum of the data, followed by post hoc analysis running Tukey’s method to test for a difference of means to a significance level of 0.05. When a data set was normally distributed, a parametric one-way ANOVA was carried out. This was followed by an all-pairwise comparison of means based on the Holm–Sidak method to a significance level of 0.05. If the statistical tests confirmed any significant difference, this was indicated as vertical bars in all plots shown, including an asterisk to indicate the magnitude as follows: * = *p* < 0.05, ** = *p* < 0.01, *** = *p* < 0.005. Any absence of such indicators is equivalent to “no significant difference confirmed”.

## 4. Results

### 4.1. Maximum Ca^2+^ Saturated Force Is Diminished in Permeabilized Single Fibres in an Oxidizing Environment

**Figure 2:**
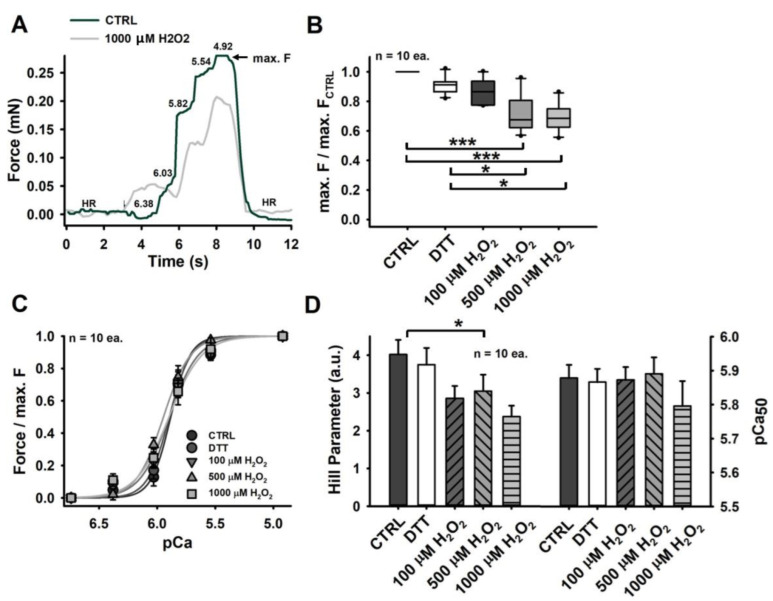
Maximum active forces decline with increasing H_2_O_2_ concentration in each single recording sequence. (**A**) example recording of a pCa-force curve at increasing Ca^2+^-concentrations (decreasing pCa). (**B**) maximum force (at pCa = 4.92) declined significantly for fibres exposed to 500 and 1000 µM H_2_O_2_, while a 60 s DTT incubation and exposure to low levels of H_2_O_2_ (100 µM) had no significant effect in relation to control levels. DTT seemed to present with forces similar to CTRL levels but was significantly larger when compared to forces produced in the presence of high concentrations of H_2_O_2_ (≥500 µM H_2_O_2_). (**C**) for each pCa level, the relative force was plotted and described by the mean reconstructed fit curve. While the inflection points (pCa_50_ value) match closely, we denote a gradual decrease in the curve’s steepness. (**D**) this decreasing steepness is reflected in a progressively declining Hill parameter, revealing a single significance between CTRL fibres and fibres exposed to 500 µM H_2_O_2_. Significance indicated as follows: * = *p* < 0.05; *** = *p* < 0.001.

Investigating the active contractility in the presence of Ca^2+^ ions in different redox states yielded a significantly reduced maximum force generation with increasing amounts of H_2_O_2_ (≥500 µM). Although a reducing environment (after 60 s of DTT incubation) and the presence of 100 µM H_2_O_2_ also seemed to slightly reduce contractile performance, their effects were not confirmed to be significant (Figure 2B).

According to our observations, this decline in contractile force does not correlate with reduced Ca^2+^ sensitivity of the contractile apparatus, which was consistently unaltered in all differently imposed redox states. This can be seen in Figure 2C, where all pCa-force curves matched very closely (similar inflection points and, thus, similar pCa_50_ values). The only marked difference we could observe was a gradual decrease in the curves’ steepness, which is reflected by a reduced Hill parameter, as shown in Figure 2D, displaying a single significant difference between the CTRL fibres and the fibres exposed to 500 µM H_2_O_2_ (roughly 30% less than for CTRL levels).

Please note that the pCa-force relationship is explicitly given in force values, as the most common notation. The corresponding stress values are given in Table 2. Additionally, note that any prior conversion from force to stress would not affect the well-known and routinised analysis procedure applied here.

### 4.2. An Oxidizing Redox Balance Imposes a Non-Linear Fibre Stiffening onto Its Passive Strain Resistance

**Figure 3:**
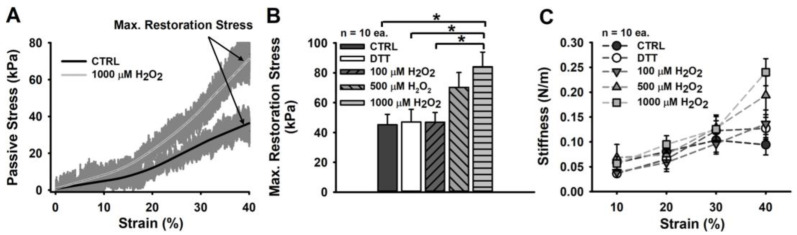
Passive restoration stress increases for larger H_2_O_2_ concentrations. (**A**) average stress–strain curve for all CTRL fibres and all fibres exposed to 1000 µM H_2_O_2_. Maximum restoration stress is obtained at 40% strain. (**B**) while DTT and exposure to 100 µM H_2_O_2_ had no marked effect on maximum restoration stress, high levels of H_2_O_2_ (1000 µM) present with a significant increase over CTRL, DTT and 100 µM H_2_O_2_ in fibres. Particularly, 1000 µM H_2_O_2_ evoked twice the restoration stress in comparison to CTRL fibres. (**C**) axial stiffness ranged between 0.05 and 0.25 N/m, with CTRL fibres displaying the least increase from 0.05 N/m to 0.1 N/m, only. All other conditions revealed a marked gradual decrease in compliance and thus, a substantial increase in stiffness at higher strains (≥30%). Significance indicated as follows: * = *p* < 0.05.

In contrast to what we observed for the active contractile properties, large concentrations of H_2_O_2_ correlate with an increased passive axial stiffness in permeabilized single muscle fibres. As seen in Figure 3B, maximum restoration stress at 40% strain appears to double for fibres exposed to ≥500 µM H_2_O_2_, which was supported by statistical analysis.

The observed stiffening under oxidizing conditions is reflected by a strong, non-linear increase that can be seen in the average stress–strain plot (Figure 3A). Here, particularly fibres exposed to 1000 µM H_2_O_2_ (light grey curve) display a much more exponential incline commencing at strain levels of ∼20% than the CTRL fibres. Exposure to the reducing agent DTT had no marked effects on passive restoration stress. 

The non-linear stiffening, which is much more apparent under oxidizing conditions, was likewise confirmed by stiffness analysis, performed by applying linear fits to each section of 10% strain (Figure 3C). Here, the entity of the samples presents a gradual but constant stiffness increase. However, the fibres exposed to ≥500 µM H_2_O_2_ strongly deviate from this relationship, displaying notably enlarged stiffness values above 20–30% strain. Such effects were not detectable for CTRL fibres, fibres exposed to DTT, or immersed in a mildly oxidizing environment (100 µM H_2_O_2_).

## 5. Discussion

### 5.1. Reduced Maximum Ca^2+^ Activated Force Does Not Originate from an Altered Ca^2+^ Sensitivity Inpermeabilized Muscle Fibres

Intact muscle fibres, exposed or subjected to H_2_O_2_ (100–500 µM) present with reduced tetanic force, which is suggested to arise from Ca^2+^ desensitization [28,29]. Intriguingly, the maximum Ca^2+^-activated force generation in permeabilized muscle fibres was also found to be reduced, however, entirely independent from the fibre’s Ca^2+^ sensitivity being unchanged [3,30]. This is likewise reflected in our analysis of active single-fibre biomechanics recordings being carried out in a more robust and highly automated robotics system. Particularly for larger H_2_O_2_ concentrations (≥500 µM), we observed a significant reduction in the maximum Ca^2+^-activated force (Figure 2B). Overall, our mean absolute force value of ∼0.26 mN for CTRL fibres matches well with maximum Ca^2+^ saturated force levels of other studies [31,32]. A conversion to stress, assuming a circular cross-section and taking the average CTRL fibre diameter of 44.3 µm into account, yields 170 kPa—a reasonable stress value for murine EDL single fibres (∼200 kPa [33]) that also translates well to the whole muscle (∼300 kPa [34]), still containing their ECM. Similar to Plant et al. (2000) and Lamb and Posterino (2003) [3,30], we also detected no change in Ca^2+^ sensitivity (pCa_50_)and observed values ranging from 5.8 to 5.9 in our experiments. In additional agreement with the literature, our Hill parameter likewise declined by over 30% (Figure 2D).

The diverse findings in intact muscle fibres (tetanic force reduced due to the Ca^2+^ desensitization of the contractile apparatus) and permeabilized fibres (Ca^2+^ activated force reduced without altered Ca^2+^ sensitivity) suggest that ROS influence force generation via a pathway that is disrupted when the muscle fibre is permeabilized [1]. This would involve a cytosolic protein as a key player, which, after skinning, is replaced by an externally applied solution. This theory finds support in studies involving the reducing agent DTT, which is capable of ‘*shielding*’ the fibre against aggressive, oxidizing radicals [1,3,4]. DTT is a membrane-permeant thiol donor that reverses disulphide bonds to regenerate reduced thiol (-SH) moieties. DTT was shown to reverse the Ca^2+^-desensitizing effects of H_2_O_2_ in intact muscle fibres [1,4]. Intriguingly, this ‘*protective*’, or sometimes even described as ‘*preventive*’ [1] effect, appears to be absent in permeabilized fibres—as shown by others [3] and confirmed by our study in a highly automated system. In this setting, the *MyoRobot* allowed us to reproduce previous findings on the Ca^2+^-responsiveness of the contractile apparatus with ease, in due time, and in an automated sequence that can be carried out consecutively without interchanging the sample. While the findings from previous studies contribute valuable knowledge, often with sophisticated self-tinkered systems, many of them suffered from mentionable specimen losses within their setups or during their procedure (e.g., over 50% of fibres in [28]). Others needed to *estimate* important structural parameters, e.g., fibre diameter, due to engineering shortcomings [30]. These challenges must have tremendously prolonged experiment time and required tight supervision, ranking even some of the most extensive biomechanics studies on redox changes in the order of having processed less than ten samples [3,10,30]. Within two weeks, after concluding the experiment design and performing protocol optimizations and adaptations, we were able to record 10 entire data sets from single muscle fibres for all mentioned conditions with our *MyoRobot* (two–three mouse legs each week, two–three single fibres recorded each day, each recording sequence taking approx. 3 h). This confirms that our technology is a resourceful tool for investigating the redox-related changes in active force generation (e.g., the Ca^2+^-sensitivity of the contractile apparatus) in permeabilized muscle fibres with ease.

### 5.2. An Oxidized Redox Balance Promotes Increased Stress with Strain in Permeabilized Muscle Fibres

In contrast to multiple studies that assessed the active biomechanics of skeletal muscle fibres at different redox states, to the best of our knowledge, an assessment of the fibre’s passive mechanical properties has neither been initiated nor performed. This likely originates from the technical complexity and precision–actuation requirements that are neither met by commercial single-fibre biomechanics systems nor by most custom-engineered devices. However, our *MyoRobot* is well-capable of performing such experiments via extremely slow, truly linear stretches that minimize any viscous relaxation [20]. The passive stiffness of muscle (fibres) plays an important role in many human diseases and conditions that are accompanied by increased ROS activity and decreased muscle function, such as Duchenne Muscular Dystrophy [35,36], fibrosis [37,38,39], sepsis [40,41], and cancer cachexia [42,43].

In the literature, H_2_O_2_ is described as a potential modulator of cysteine and methionine residues, acting as an important redox sensor in selected proteins [44]. Given the proposed role of methionine [9,18] and cysteine [45], it is plausible that both contribute to protein function and may constitute a mechanism for protein regulation [46]. Under oxidizing conditions, methionine is oxidized to methionine sulfoxide through the addition of an oxygen atom to its side chain. According to Hoshi et al. (2001), this increases the stiffness and the polarity of the molecule, potentially augmenting dipole–dipole forces [46].

In addition, methionine sulfoxide is involved in the conversion from catalytic cysteine, in its protonated form (Cys-SH), to cysteine sulfenic acid (Cys-SOH) via a reductase (see [47], Figure 1) [47]. The presence of both forms then allows the formation of strong disulphide bonds that, in vitro, can only be reversed by an external reducing agent (e.g., DTT) [47]. As such, the prolonged effect of H_2_O_2_ on two of its potential targets (cysteine and methionine) would suggest a marked increase in passive restoration stress through additionally introduced disulphide bonds.

Interestingly, this idea finds support in our results, postulating an almost two-fold, significant increase in passive restoration stress of samples exposed to 1000 µM H_2_O_2_ over CTRL single fibres (Figure 3B). This effect was already seen at 500 µM H_2_O_2_, yet, in a less pronounced fashion, while lower concentrations or reducing conditions (after 60 s incubation in DTT) imposed no detectable change in passive restoration stress at 140% of the fibre’s resting length (SL: ∼3.2 µm). The here-reported maximum stress values of ∼40 kPa for the CTRL fibres are slightly larger than the values obtained from comparable experiments on murine EDL (e.g., ∼30 kPa at ∼3.2 µm SL [48], and ∼21 kPa at 40% strain [20]), yet, still approx. half the stress reported in *M. soleus* single fibres (∼60 kPa [49]). The latter are known to display twice the stiffness of EDL single fibres in rats [50] and similarly in mice [27].

The marked increase in stiffness observed under highly oxidizing conditions (≥500 µM H_2_O_2_) was also expressed by an exponentially increasing stiffness and resistance to strain (restoration force development in response to elongation changes) (Figure 3C), while the CTRL fibres displayed a more linear relationship here. Similar effects were detected when comparing single muscle fibres-to-fibre bundle preparations, with the latter still being surrounded by an extracellular matrix. While single fibres present with an almost linear stress–strain relationship, the presence of an extracellular matrix in fibre bundles contributed to rather exponential stress–strain relationships and, thus, non-linear effects [48,51,52]. As such, we hypothesize that a highly oxidizing environment promotes a non-linear stress–strain behaviour through a potentially reduced linear elastic stretch regime. However, if such a mechanism can solely be attributed to disulphide bond formation through ROS acting upon cysteine and methionine residues or if it involves post-translational modifications to structural proteins, e.g., actin [53] or titin [54,55,56], remains to be addressed in future studies.

In that regard, a previous study on mechanically ventilated rats in an ICU model associated with increased ROS production has confirmed the presence of multiple post-translational modifications of the myosin-heavy chain [57]. Perspectively, such protein modifications could be directly (e.g., ROS oxidation of regulatory sulfhydryls [58]) or indirectly imposed (e.g., phosphorylation via redox-sensitive kinases [53]). However, it remains to be clarified if the major targets of either potential mechanism truly align with the proteins that would normally determine passive stiffness to a major extent (e.g., titin [59,60], connectin [61], desmin [31], etc.).

## 6. Conclusions

We provide the first data that examine the passive elastic properties in the form of stress–strain curves performed in single, permeabilized muscle fibres at an oxidizing redox balance. Therefore, our study presents the first link between active and passive biomechanics properties in the field of muscle redox biology. By detecting a significant decrease in maximum Ca^2+^ activated force in an oxidizing environment, e.g., the presence of H_2_O_2_, we could easily reproduce data from previous studies. In addition, we observed an increased passive axial stiffness, significantly enhanced in the fibres exposed to larger concentrations of H_2_O_2_ (≥500 µM), which supports a potentially new mechanism for passive stiffness modulation that can be of clinical relevance. However, it is still unclear whether these findings may arise from a rising formation of disulphide bonds on certain amino acids in an oxidizing environment or post-transcriptional modification to structural proteins, which remains to be addressed in future experiments. Nevertheless, our *MyoRobot* is capable of delivering passive stress–strain data with ease in feasible and repetitive experiments and, thus, will provide a resourceful tool to study redox balance biomechanics in muscles.

## Figures and Tables

**Table 1:** Setup of bioactive solutions in the *MyoRobot* rack.

Well	Bioactive Solution	Purpose
1	Saponin (0.01% *w*/*v*)	fibre skinning
2	LR	replace EGTA with HDTA, relax
3	HR	buffer excess Ca^2+^, relax
4	pCa 6.38	initiate contraction
5	pCa 6.03	initiate contraction
6	pCa 5.82	initiate contraction
7	pCa 5.54	initiate contraction
8	pCa 4.92	initiate maximum contraction
9	DTT	reducing environment
10	LR (100 µM H_2_O_2_)	replace EGTA with HDTA, relax
11	HR (100 µM H_2_O_2_)	buffer excess Ca^2+^, relax
12	pCa 6.38 (100 µM H_2_O_2_)	initiate contraction
13	pCa 6.03 (100 µM H_2_O_2_)	initiate contraction
14	pCa 5.82 (100 µM H_2_O_2_)	initiate contraction
15	idle	relaxing environment
16	pCa 5.54 (100 µM H_2_O_2_)	initiate contraction
17	pCa 4.92 (100 µM H_2_O_2_)	initiate maximum contraction
18	LR (500 µM H_2_O_2_)	replace EGTA with HDTA, relax
19	HR (500 µM H_2_O_2_)	buffer excess Ca^2+^, relax
20	pCa 6.38 (500 µM H_2_O_2_)	initiate contraction
21	pCa 6.03 (500 µM H_2_O_2_)	initiate contraction
22	pCa 5.82 (500 µM H_2_O_2_)	initiate contraction
23	pCa 5.54 (500 µM H_2_O_2_)	initiate contraction
24	pCa 4.92 (500 µM H_2_O_2_)	initiate maximum contraction
25	LR (1000 µM H_2_O_2_)	replace EGTA with HDTA, relax
26	HR (1000 µM H_2_O_2_)	buffer excess Ca^2+^, relax
27	pCa 6.38 (1000 µM H_2_O_2_)	initiate contraction
28	pCa 6.03 (1000 µM H_2_O_2_)	initiate contraction
29	pCa 5.82 (1000 µM H_2_O_2_)	initiate contraction
30	pCa 5.54 (1000 µM H_2_O_2_)	initiate contraction
31	pCa 4.92 (1000 µM H_2_O_2_)	initiate maximum contraction

**Table 2:** Comparison of maximum contractile stress produced in a Ca^2+^ saturated environment and maximum restoration stress in response to strain. Significance indicated as follows: * = *p* < 0.05 vs. CTRL, and # = *p* < 0.05 active vs. passive.

Stress (kPa)	CTRL	DTT	100 µM H_2_O2	500 µM H_2_O_2_	1000 µM H_2_O_2_
**Contractile** **Restoration** **stress at 140% L**0 **(∼3.2 µm SL)**	172 ± 20 # 45 ± 7 #	156 ± 19 # 47 ± 8 #	148 ± 17 # 47 ± 6 #	130 ± 19 *# 70 ± 9 #	118 ± 14 *# 84 ± 9 *#

## Data Availability

The data that support the findings of this study are available from the corresponding author, M.H., upon request.

## Abbreviations

The following abbreviations are used in this manuscript:

EDL	M. Extensor Digitorum Longus
CTRL	Control
FT	Force Transducer
VC	Voice Coil
HA	High Activating (Solution)
HR	High Relaxing (Solution)
LR	Low Relaxing (Solution)
HKS	High-Potassium Solution
EDTA	Ethylene glycol-bis(β-aminoethylether))-N,N,N′,N′-tetraacetic acid
HDTA	Hexamethylenediaminetetraacetic acid
DTT	Dithiothreitol
